# Exploring the steps of learning: computational modeling of initiatory-actions among individuals with attention-deficit/hyperactivity disorder

**DOI:** 10.1038/s41398-023-02717-7

**Published:** 2024-01-08

**Authors:** Gili Katabi, Nitzan Shahar

**Affiliations:** 1 School of Psychological Sciences, Tel Aviv University, Tel Aviv, Israel; 2 Sagol School of Neuroscience, Tel Aviv University, Tel Aviv, Israel

**Keywords:** ADHD, Learning and memory, Human behaviour

## Abstract

Attention-deficit/hyperactivity disorder (ADHD) is characterized by difficulty in acting in a goal-directed manner. While most environments require a sequence of actions for goal attainment, ADHD was never studied in the context of value-based sequence learning. Here, we made use of current advancements in hierarchical reinforcement-learning algorithms to track the internal value and choice policy of individuals with ADHD performing a three-stage sequence learning task. Specifically, 54 participants (28 ADHD, 26 controls) completed a value-based reinforcement-learning task that allowed us to estimate internal action values for each trial and stage using computational modeling. We found attenuated sensitivity to action values in ADHD compared to controls, both in choice and reaction-time variability estimates. Remarkably, this was found only for first-stage actions (i.e., initiatory actions), while for actions performed just before outcome delivery the two groups were strikingly indistinguishable. These results suggest a difficulty in following value estimation for initiatory actions in ADHD.

## Introduction

Attention-deficit/hyperactivity disorder (ADHD) is a neurodevelopmental disorder that is characterized by inattention, impulsivity, and hyperactivity [1, 2]. Approximately 10% of school-aged children are affected by ADHD [3], with the majority experiencing significant symptoms throughout adolescence and into adulthood [4]. Individuals with ADHD frequently struggle with the completion of tasks that require the execution of multiple actions (e.g., doing homework) [5–7]. Despite the known difficulties experienced by those with ADHD in completing multi-action tasks in natural environments, there has been no prior investigation of ADHD in the context of value-based sequence learning. In this study, we employed reinforcement learning modeling to examine the extent to which ADHD is associated with reduced value-based learning for initial actions (i.e., actions at the start of the sequence) using a multi-stage reinforcement learning task.

Reinforcement learning studies have demonstrated that human individuals estimate the value of potential actions based on their outcome history [8–10]. This estimation process enables individuals to act persistently, even when the overall goal or outcome is distant [11, 12]. Specifically, reinforcement learning algorithms suggest that when making a sequence of actions that lead to a certain outcome, the value of the observed outcome is mentally “backpropagated” to preceding actions [13]. For example, when making a cup of coffee, multiple actions must be completed before achieving the end goal (i.e., consumption of the beverage). These actions should be associated with the value of the reached outcome, thereby increasing the likelihood of repeating/avoiding the sequence based on the value of the outcome. The backpropagation of values to actions is considered a “glue” that binds the actions into a sequence [14]. However, during the backpropagation process, value is also discounted across the sequence, meaning that initiatory actions (i.e., actions performed at the start of a sequence) receive smaller value updates than terminal actions (i.e., actions performed immediately before outcome delivery) [10, 15]. The challenge is that without making correct value-based choices early in the sequence, the end goal cannot be reached, resulting in less consistent and more arbitrary value-based choices overall. Nevertheless, since initiatory actions are distant in time from the outcome, they are also assigned smaller value updates [10]. Many studies have already suggested that the processes described by reinforcement learning equations do indeed predict human behavior [15–17] and can explain neural activity [9, 18–22]. However, no study that we know of to date has used this approach to investigate whether the value-based process can explain the difficulty of participants with ADHD to engage and maintain a multi-stage task in order to reach a desired goal.

Here, we hypothesize that difficulties acting according to obtained rewards in ADHD will be more pronounced in initiatory actions (i.e., actions that are performed at the start of a sequence) compared with terminal actions (i.e., actions that are performed at the end of an sequence). Specifically, we suggest that the process of updating the value of initiatory actions is less efficient in ADHD, leading to noisier choices at the start of a sequence. Difficulty holding and following distinct action values at the beginning of a sequence can lead to less consistent choices and difficulty achieving the desired goal, which is highly relevant to ADHD symptomatology. For example, if the initiatory action of a sequence for making a beverage is not clearly defined, other competing actions that are closer to the outcome and assigned with similar values might take precedence. Alternatively, if the value of the initiatory action is internally well defined, it will lead more frequently to the selection of the best action, generating a more consistent and uninterrupted sequence of actions.

Although no studies have examined action–outcome associations in ADHD for initiatory actions, several findings in the literature support this hypothesis. First, ADHD individuals exhibit marked differences in reward-related neural processing [23–30]. Second, ADHD individuals are known to show steeper temporal discounting of future values [31–33]. Furthermore, ADHD shows inconsistent behavior when performing sustained attention/executive function tasks without immediate reward or feedback [34–40]. Finally, when sustained attention/executive function tasks are performed under conditions of immediate reward (where every action can be considered a terminal action, immediately leading to reward), ADHD shows marked improvements [41]. Therefore, there is reason to believe that individuals with ADHD have a reduced ability to assign and follow internal action values, mostly for actions that are at the beginning of a sequence and distance in time from outcome delivery. However, no empirical investigation has directly addressed this question.

The current study, therefore, examined to what extent individuals diagnosed with ADHD were able to update and act upon action–outcome associations across different stages of a sequential decision-making task. Specifically, we hypothesized that the value-based behavior of ADHD participants would be more inconsistent (noisier choices and higher RT variability) compared with their healthy control (HC) peers, mostly for initiatory but not terminal actions. We further hypothesize that the reason for such group differences will be due to reduced action value updating for outcome-distant stages in ADHD. To examine this question empirically, ADHD/HC participants performed a sequential reinforcement learning task. In each trial, they were asked to make three choices to gain rewards. We then used computational modeling to assess the internal value each individual might be assigning for each action in each stage. We replicated previous findings showing that for all participants and across both groups, choice accuracy, and RT variability estimates improved when the latent action values were more differentiated (larger internal value difference between the two available actions). We then examined the ability of ADHD individuals to act according to the reward history across stages of the task.

## Methods

### Participants

Fifty-four undergraduate Tel-Aviv University students completed the study in return for course credits or monetary compensation. (For demographics including gender, age, and IQ, see Table 1; all participants were white caucasian; 53 came from the Jewish sector and 1 from the Arab–Christian sector). All participants reported normal or corrected vision and signed informed consent before participating in the study. Participants using ADHD medications were asked to abstain for at least 24 hours before the lab session. The study was approved by the Tel-Aviv University IRB ethics committee. Exclusion criteria included any neurological or psychiatric history past or current, history of significant head injury, and current drug or alcohol abuse. Specifically, nine participants were excluded from the study due to a concurrent diagnosis of additional disorders, including anxiety, depression, and OCD, while an additional five participants were excluded because they reported ongoing psychiatric treatment and two additional participants were excluded due to drug abuse.

### ADHD diagnosis

To ensure ADHD diagnosis (present for the ADHD group, absent for the control group), we performed the following steps: (1) we made sure that all ADHD participants received an ADHD diagnosis in the community by a licensed mental health professional (approved by the Israeli Government Health Department in making ADHD diagnosis). (2) We made sure that no participants in the control group received an ADHD diagnosis in the community. (3) We questioned the participants in both groups to ensure they were not diagnosed in the community with any additional mental health disorder (current or past). (4) All participants completed a well-validated ADHD assessment in our lab that included a semi-structured interview using the Diagnostic Interview for ADHD in adults (DIVA [42]) to reconfirm the presence/absence of an ADHD diagnosis for the clinical/control group. DIVA assessments were conducted by well-trained psychology students under the direct supervision of a licensed clinical psychologist expert in ADHD. (5) All participants completed self-report questionnaires for ADHD symptoms in adulthood (ASRS [43]) and childhood (WURS [44]) to provide an estimate for the difference of ADHD symptoms between the groups in our cohort (see Table 1 and Fig. S1). (6) To further describe our cohort and examine group differences in other relevant estimates, participants completed an IQ test (Ravens [45]), self-report estimates for symptoms of depression (beck depression inventory; BDI [46]), anxiety (state trait anxiety inventory; STAI [47]), obsessive–compulsive (obsessive–compulsive inventory; OCI [48]), autism (autism spectrum quotient; AQ [49]), alcohol use disorder (Alcohol Use Disorders Identification Test, AUDIT [50]) and cannabis use disorder (Cannabis Use Disorders Identification Test, CUDIT [51]). Overall, we found no statistically significant group difference in these scores, while we did find a substantial group difference in ADHD self-report estimates (ASRS and WURS), as should be expected.

**Table 1:** Characteristics of ADHD and Healthy control groups.

	Healthy control	ADHD	*p*-Value
*N*	26 (female: 20)	28 (female: 15)	
Age	19–34 (SD = 4.30)	20–32 (SD = 2.68)	0.38
ASRS score	38.2 (SD = 10.5)	61.7 (SD = 6.6)	<0.001
WURS score	20.0 (SD = 18.5)	44.4 (SD = 13.1)	<0.001
OCI score	40.6 (SD = 28.1)	32.3 (SD = 23.6)	0.25
STAI score	41.6 (SD = 9.6)	45.4 (SD = 9.9)	0.16
BDI score	9.1 (SD = 9.7)	11.3 (SD = 7.2)	0.35
AQ score	15.9 (SD = 5.6)	16.9 (SD = 5.8)	0.44
Raven score	12.2 (SD = 3.4)	12.5 (SD = 3.7)	0.76
AUDIT	2.5 (SD = 2.95)	2.88 (SD = 1.36)	0.62
CUDIT	4.61 (SD = 6.07)	3.23 (SD = 3.32)	0.41
Stimulants Medication	–	10—Methylphenidate based 10—Amphetamine based	

Note. *ASRS* adult ADHD self-report scale, *WURS* Wender Utah rating scale for attention deficit hyperactivity, *OCI* obsessive-compulsive inventory, *STAI* state-trait anxiety inventory, *BDI* Beck depression inventory, *AQ* autism spectrum quotient, *Raven* Raven progressive matrices, *AUDIT* alcohol use disorders identification test, *CUDIT* Cannabis use disorders identification test. Note that CUDIT and AUDIT scores were obtained after the study’s completion (as a response to a reviewer’s comment) and thus included 18/17 participants from the HC/ADHD group, respectively. All other measurements were completed by all participants included in study.

### Reinforcement learning task

**Fig. 1:**
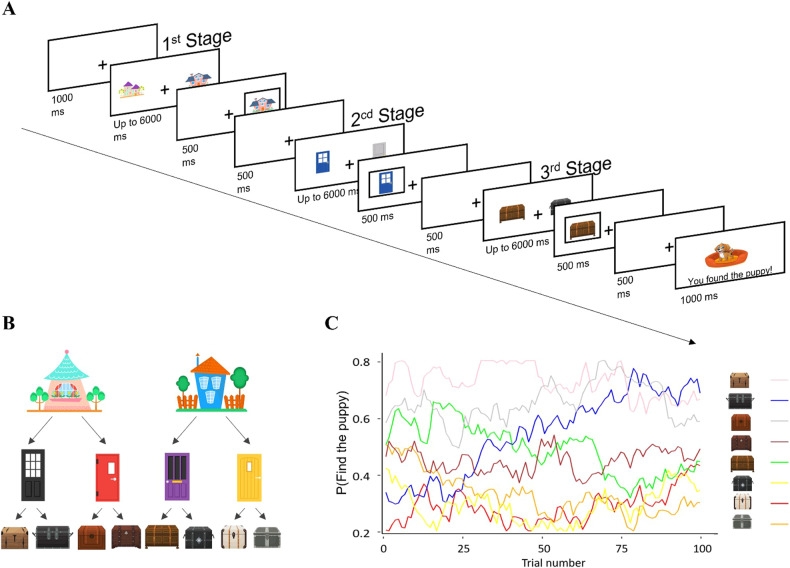
Trial sequences for the multiple-stage task. Participants were told that a puppy was hiding behind a chest and that their task was to locate the puppy. **A** Each trial individuals made choices across three stages (houses, doors, and chests) to try and locate the puppy. **B** State-action transition structure shows a deterministic transition from stage one (houses), stage two (doors), and stage three (chests). **C** We used a typical reinforcement learning design where the true expected value (probability of finding the puppy for each chest) for each chest drifted slowly across the block, thus requiring participants to keep learning across the task, similar to conventional reinforcement-learning paradigms [10, 94–97].

Participants performed a multiple-stage reinforcement learning task (Fig. 1) that included three sequential stages. The participants were told that their task was to find a puppy that was hiding behind different chests and that they needed to make three choices each trial to try and locate the puppy. They were told that the task included two houses, each house containing two rooms and each room containing two chests. Accordingly, at the first stage of each trial, participants chose one of two “houses” (denoted by cartoon houses stimuli, see Fig. 1B), each leading them deterministically to a second-stage state where a pair of “doors” (cartoon stimuli, see Fig. 1B) was offered. Choosing one of the doors led participants deterministically to a third-stage state where a pair of “chests” (cartoon stimuli; see Fig. 1B) was offered. After making all three choices, the participants were presented with the outcome, which was a cartoon image of a happy puppy or an empty pet pillow with no puppy (see Fig. 1A). Specifically, each chest delivered a reward (finding the puppy) on a drifting probability (see Fig. 1C), and participants were asked to learn which house, door and chest are most likely to lead to reward throughout the task. (For more information, see SI).

### Choice accuracy and difficulty

For the purposes of analysis, we wanted to code choice accuracy and choice difficulty for each trial and stage. In each stage, participants were asked to make a choice between two actions. A choice was coded as “correct” if the participant selected the action that took part in the sequence with the highest true expected value at a certain stage. Choice difficulty was defined in a similar way, according to the true expected values of the sequence associated with each offered option. (For more information, see SI).

### Using ex-Gaussian distribution to estimate RT variability

An ex-Gaussian distribution is a non-theoretical statistical model that was extensively used in cognitive [52] and ADHD research [53–55] to describe the distribution of reaction times. It combines two different distributions: the Gaussian (normal) distribution, which represents the more frequent and typically quicker reaction time, and an exponential distribution, which represents the tail of slower reaction times. Specifically, *τ* is the parameter that describes the exponential portion of the distribution. By using the ex-Gaussian distribution, researchers can more accurately model the full range of reaction times, including the long tail of slower responses. (For more information, see SI).

### Data treatment

The first trial in each block, trials with implausibly quick reaction times (<200 ms) or exceptionally slow reaction times (>4000 ms) were omitted (5.15% of all trials). Participants with more than 25% excluded trials (one participant, HC group) or higher than 5% no response rate (none), were further excluded from the analysis. Due to technical issues, we found after data collection that some participants did not receive choice feedback on a minority of trials. Those trials were omitted (69 trials overall), and participants with more than 5% of omitted trials were excluded (4 participants, 3 participants from the HC group, and 1 participant from the ADHD group).

### Computational modeling

To estimate the internal values participants might assign to each available action, we fitted a well-established hierarchical reinforcement learning model to participants’ choice behavior [16, 56, 57]. First, we defined a prediction error (PE) for each state and action according to its expected value and the observed rewards as follows (see Eqs. 1–3):

1 $${\rm{PE}}_{1}={V}_{\rm{choice}2}-{V}_{\rm{choice}1}$$

2 $${\rm{PE}}_{2}={V}_{\rm{choice}3}-{V}_{\rm{choice}2}$$

3 $${\rm{PE}}_{3}=R-{V}_{\rm{choice}3}$$

Where *V*_choice1_, *V*_choice2,_ and *V*_choice3_ are the latent state-action values for the chosen actions in each state (in the reinforcement learning literature, also known as *Q* values). *R* refers to the outcome observed following the terminal state so that *R* ∈ {0,1}. We followed the convention in model-free reinforcement learning modeling [10], where each PE is backpropagated and assigned to all past actions under a discounting factor (i.e., free parameter). Therefore, the first action is updated using PE_1_, PE_2_, and PE_3_. The second action is updated using PE_2_ and PE_3_. The third action is updated using PE_3_. Thus, the value of every action was updated as follows (see Eqs. 4–6):

4 $$\begin{array}{l}{V}_{\rm{choice}1}={V}_{\rm{choice}1}+{\alpha }_{1}\left[{V}_{\rm{choice}2}-{V}_{\rm{choice}1}\right]+{\alpha }_{1}\lambda \left[{V}_{\rm{choice}3}-{V}_{\rm{choice}2}\right]\\\qquad\qquad+\,{\alpha }_{1}{\lambda }^{2}[R-{V}_{\rm{choice}3}]\end{array}$$

5 $${V}_{\rm{choice}2}={V}_{\rm{choice}2}+{\alpha }_{2}\left[{V}_{\rm{choice}3}-{V}_{\rm{choice}2}\right]+{\alpha }_{2}\lambda [R-{V}_{\rm{choice}3}]$$

6 $${V}_{\rm{choice}3}={V}_{\rm{choice}3}+{\alpha }_{3}[R-{V}_{\rm{choice}3}]$$

Where *α*1, *α*2, and *α*3 are the learning rates for Stages 1, 2, and 3, respectively *λ* is an eligibility trace factor referring to the extent of prediction error backpropagation. Specifically, for *λ* of 1, the agent will learn and update whole sequences, while for *λ* = 0 the agent will learn individual state action values for each stage. Finally, choices were predicted using a softmax decision policy (see Eqs. 7–9):

7 $$p\left({\rm{choice}}1\right)=\frac{\exp \left({\beta }_{1}{V}_{\rm{choice}1}\right)}{\Sigma \exp \left({\beta }_{1}{V}_{1}\right)}$$

8 $$p\left({\rm{choice}}2\right)=\frac{\exp \left({\beta }_{2}{V}_{\rm{choice}2}\right)}{\Sigma \exp ({\beta }_{2}{V}_{2})}$$

9 $$p\left({\rm{choice}}3\right)=\frac{\exp \left({\beta }_{3}{V}_{\rm{choice}3}\right)}{\Sigma \exp ({\beta }_{3}{V}_{3})}$$

where *β*1, *β*2, and *β*3 are the decision temperature for Stages 1, 2, and 3, respectively. Moreover, to ensure that the model can be used adequately to estimate latent parameters, we performed a parameter recovery analysis, which indicated excellent parameter recovery with no indication of bias (see Fig. S3; for more information, see SI).

## Results

### Theory-independent analysis

#### Accuracy rates—group difference analysis

**Fig. 2:**
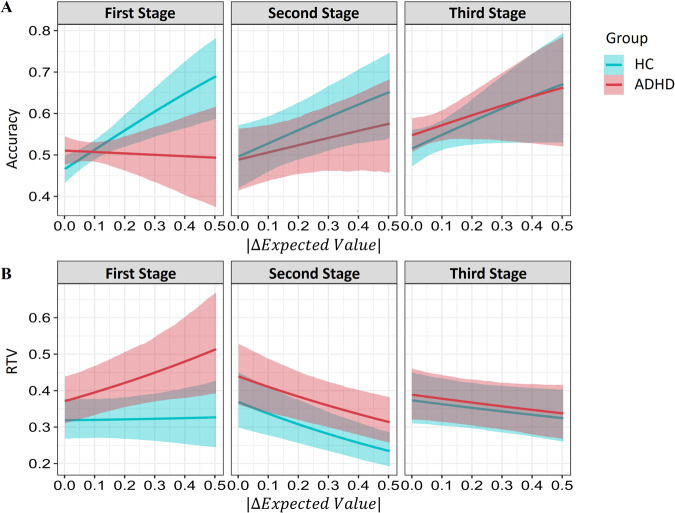
Effect of choice difficulty on accuracy rates and reaction-time variability. **A** The influence of choice difficulty, group (HC vs. ADHD), and stage (1, 2, or 3) on participants’ accuracy rates. First, results demonstrate that across stages and groups, accuracy rates improve as a function of choice difficulty. Importantly, ADHD individuals showed an attenuated sensitivity to choice difficulty changes only in the first stage but not in the second and third stages. **B** The influence of choice difficulty, group, and stage on participants’ RT variability. Results demonstrate that across stages and groups, RT variability improved when choices were easier. Importantly, ADHD individuals showed an attenuated sensitivity to choice difficulty changes only in the first stage (*y*-axis represents log(*τ*) estimates for an ex-gaussian distribution fitted to empirical data).

To examine the difference in performance between ADHD and HC participants on accuracy rates, we performed a hierarchical Bayesian regression analysis (for more details, see SI) where we predicted choice accuracy (0/1 for choosing the offer with the lower/higher true expected value, respectively), using choice difficulty (absolute difference between the true expected values of the two offers, see Methods), stage (1, 2, or 3) and group (HC vs. ADHD). When examining the interaction between group and choice difficulty, we found that the effect of choice difficulty on accuracy rates was smaller for ADHD compared to HC only for first-stage choices. Specifically, a change of 0.5 in the difference between the expected value of the two offered actions in the first stage was associated with an increase of 22.6% in accuracy rates for the HC group and only 1.8% for the ADHD group [posterior median of the groupXchoice-difficulty effect in the first stage = −2.00 CI_89%_ between −3.35 and −0.71, CI_95%_ between −3.58 and −0.48, probability of direction (pd) = 99.30; see full posterior in Fig. S5A]. Strikingly, group differences vanished completely and even changed direction when examining second and third-stage choices. Specifically, we found a tendency toward a group × choice difficulty interaction in the opposite direction to that of the first stage when examining the second stage [groupXchoice-difficulty effect posterior median = 1.45 CI_89%_ between −0.38 and 3.22, CI_95%_ between −0.80 and 3.69, pd = 89.45%; See full posterior in Fig. S5B] and the third stage [groupXchoice-difficulty median = 1.67, CI_89%_ between −0.19 to 3.58, CI_95%_ between −0.65 and 4.00, pd = 92.15%; see full posterior in Fig. S5C]. Therefore, we conclude that ADHD participants were not as sensitive to differences in choice difficulty as the HC group when making first-stage choices. However, in the second and third choices, we found no evidence for group differences in accuracy rates (see Fig. 2A). (For additional analysis regarding accuracy rates across groups and the effect of IQ on accuracy rates, see SI).

#### Reaction-time variability—group difference analysis

We next examined the effect of choice difficulty (absolute difference between the true expected values of the two offers, see Methods), group (HC vs. ADHD), and stage (1, 2, or 3) on RT variability using a hierarchical Bayesian regression analysis (for more details see SI). We found that the effect of choice difficulty on RT variability was smaller for HC compared to ADHD in first-stage choices (see Fig. 2B). Specifically, a change of 0.5 in the difference between the expected value of the two offered actions was associated with an increase of 8 ms in the tail of the RT distribution for the HC group. However, the ADHD showed 136 ms change for the same amount of difference [posterior median for group × choice-difficulty effect on *τ* estimates on the first stage = +0.60 CI_89%_ between 0.03 to 1.16, CI_95%_ between −0.08 and 1.28, pd = 95.25%; see full posterior in Fig. S6A]. Strikingly, for the second and third stages, this effect reversed with a slightly larger effect of choice difficulty on RT variability estimates for ADHD compared to HC. Specifically, the group effect on *τ* estimates was negative for both the second stage [posterior median = −0.37 CI_89%_ between −1.05 and 0.29, CI_95%_ between −1.19 and 0.43, pd = 80.00%; see full posterior in Fig. S6B] and third stage [posterior median = −0.60 CI_89%_ between −1.28 and 0.05, CI_95%_ between −1.41 and 0.24, pd = 92.35%; see full posterior in Fig. S6C; for coefficients report see Table S2]. (For additional analysis regarding reaction time variability rates across groups, see SI).

### Computational reinforcement learning modeling

The previous analysis showed that ADHD individuals were less sensitive to the difference in the true expected values of the different choices in the first stage using both choice-accuracy and RT variability estimates. However, true expected values are quantified according to the pre-defined experimental design. Since the current task is a reinforcement learning task, the reward history is not only a function of the experimental design but also a function of the participant’s choices. To track and gain a better estimate of the individual internal action value for each trial and stage (also referred to as *Q*-values in reinforcement learning), we used a well-established hierarchical reinforcement learning modeling (for details, see SI). We fitted the model to each group separately using hierarchical Bayesian parameter estimation (for details, see SI). We further performed a nested model comparison and found that a saturated seven-parameter model had the best fit to the empirical data (for details, see SI).

#### Accuracy rates estimated using internal action values

We used the expected value of the *Q*-value posterior distribution obtained for each action in our model Bayesian sampling as an estimate for the internal action values for each action (see “Methods”). To estimate choice accuracy, we first defined an accurate choice as one that reflects a selection in the higher internal value action in each stage (defined as 1 if the action with the higher internal value was taken and 0 otherwise). We repeated the same regression analyses reported in the previous section, with absolute differences in internal value (i.e., |Δ*Q*|, this notion is used due to the common notion of “Q-values” for internal values in the reinforcement learning literature) group (ADHD vs. HC), stage (1, 2, and 3) and their interactions as predictors for choice accuracy.

**Fig. 3:**
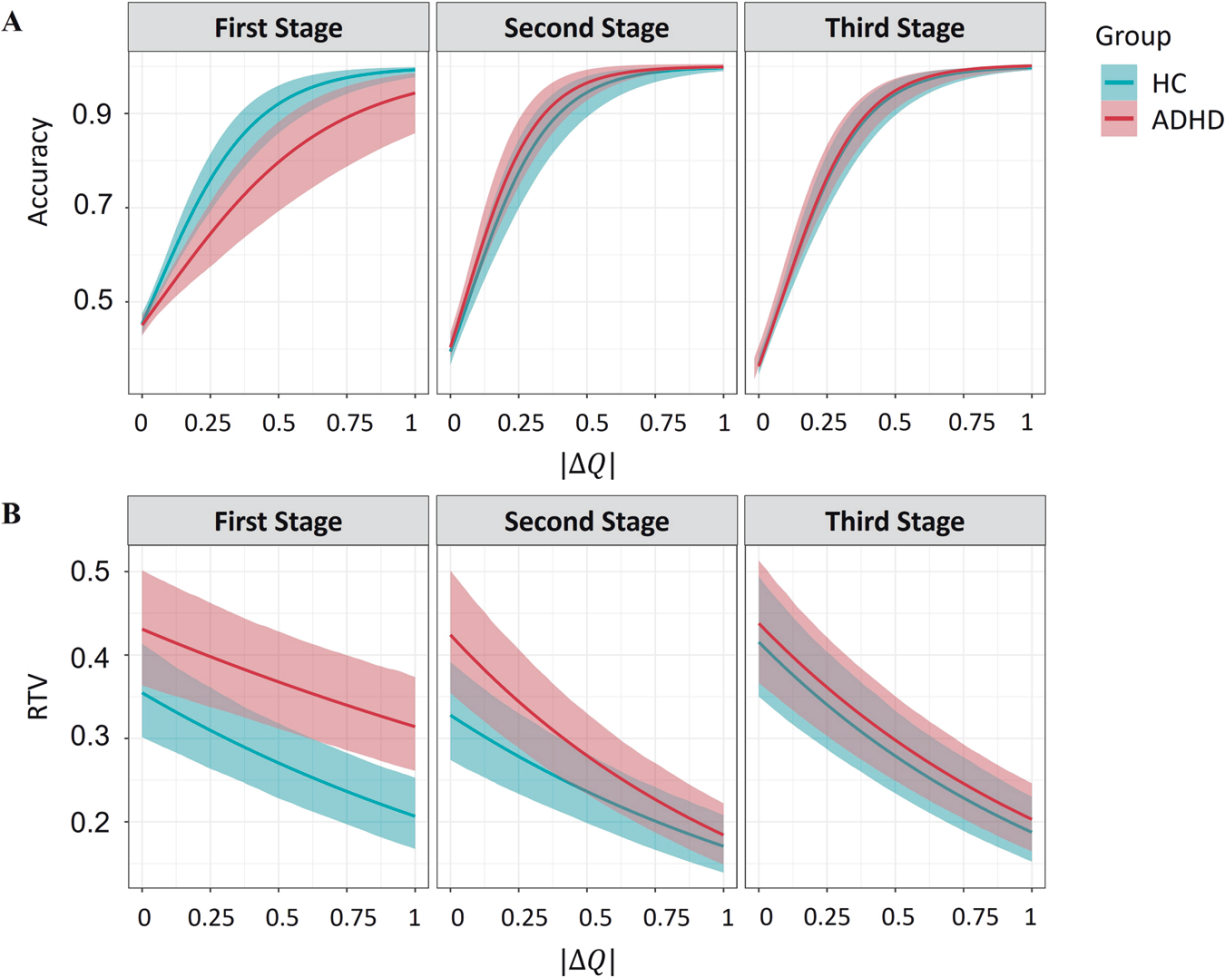
Effect of latent internal values difference for each action on accuracy rates and reaction-time variability. **A** Choice accuracy estimates as a function of group, stage, and absolute difference between the internal values estimated using a reinforcement learning model (i.e., |Δ*Q*|). **B** RT variability as a function of group, stage and absolute difference between internal action values (i.e., |Δ*Q*|). (*y*-axis represents log(*τ*) estimates for an ex-gaussian distribution fitted to empirical data).

First, we found that higher *Q*-value differences were coupled with higher choice accuracy across stages and groups [posterior median = 5.39 CI_89%_ between 4.19 and 6.50, CI_95%_ between 3.90 and 6.75, pd ~100%; see full posterior in Fig. S7A]. Specifically, a change of 0.5 in the difference between the two offered action values was associated with an increase of 51% in accuracy rates. This finding shows that the model was able to capture some estimates for values that indeed predict participants’ behavior well. Second, we further found a substantial group × |Δ*Q*| interaction for Stage 1, so that ADHD exhibited lower sensitivity to *Q*-values difference in terms of choice accuracy compared with HC [posterior median of group × |Δ*Q*| effect in the first stage = −2.19 CI_89%_ between −3.75 and −0.63, CI_95%_ between −4.06 and −0.25, pd = 98.65%; see full posterior in Fig. S7B; see Fig. 3A]. Strikingly, similarly to former results, group differences vanished completely and even changed direction when examining second and third stage choice. Specifically, we found a tendency toward a group × |Δ*Q*| interaction in the opposite direction to that of the first stage when examining the second stage [posterior median = 3.11 CI_89%_ between 1.21 to 5.13, CI_95%_ between 0.67 and 5.54, pd = 99.30%; see full posterior in Fig. S7C] and the third stage [posterior median = 2.40 CI_89%_ between 0.40 and 4.34, CI_95%_ between −0.12 and 4.82, pd = 97%; see full posterior in Fig. S7D]. Overall, these findings resonated with the finding that ADHD had lower decision noise parameters at Stage 1 compared to their peers.

#### Reaction-time variability as a function of internal action values

We sampled a hierarchical Bayesian regression where the absolute difference in *Q*-values (i.e., |Δ*Q*|) RT variability group (ADHD vs. HC), stage (1, 2, or 3), and their paired interaction as predictors for the *τ* parameter in an ex-Gaussian distribution fitted to trial-by-trial reaction-time estimates (see “Methods”). First, we found that higher *Q*-value differences were coupled with lower *τ* values across all stages [posterior median = −0.54 CI_89%_ between −0.67 and −0.41, CI_95%_ between −0.70 and −0.38, pd ~100%; see full posterior in Fig. S8A]. This finding supports the validity of our |Δ*Q*| since longer RT tails are expected for decisions with similar values [58–60]. Furthermore, our model estimates of internal action values were based only on choices, without introducing RTs to the model during parameter estimation. Thus, finding this expected association |Δ*Q*| serves as an important sanity check for our modeling approach.

Second, we found a substantial Group × |Δ*Q*| interaction for Stage 1, so that ADHD exhibited lower sensitivity to *Q*-values difference in terms of *τ* estimates [posterior median = 0.23 CI_89%_ between 0.07 and 0.38, CI_95%_ between 0.03 and 0.41, pd = 99%; see full posterior in Fig. S8B; see Fig. 3B]. However, for the second stage, this effect reversed with a significant effect of *Q*-value difference on RT variability estimates for ADHD compared to HC [posterior median = −0.41 CI_89%_ between −0.63 and −0.18, CI_95%_ between −0.68 and −0.14, pd = 99.9%; See full posterior in Fig. S8C]. For the third stage, this effect remained negative with a slightly smaller effect of *Q*-value difference on RT variability estimates for ADHD compared to HC [posterior median = −0.20 CI_89%_ between −0.42 and 0.03, CI_95%_ between −0.46 and 0.08, pd = 91.2%; see full posterior in Fig. S8D; for coefficients report see Table S4].

## Discussion

The current study examined the ability of ADHD individuals to assign and act upon internal action values across stages of a sequential decision-making task. Participants with/without ADHD made decisions across a three-step deterministic tree task. First, we used reinforcement learning modeling to gain estimates of the latent and internal action values for each individual, trial, and step. We replicated previous findings showing that across both groups, estimates of choice accuracy and RT variability improved when the two available actions were well differentiated in terms of their latent values [61, 62]. Second, we found that ADHD individuals were less sensitive to initiatory action value differences across both choice accuracy and RT variability estimates compared with their HC. Specifically, a larger difference between the values of the two available first-stage actions had smaller benefits on ADHD choices and RTs compared to HC. Remarkably, these group differences diminished and disappeared when making terminal third-stage actions to a point where the two groups were remarkably indistinguishable. Our results, therefore, suggested a striking attenuated sensitivity to value actions in ADHD, only in initiatory but not terminal task action.

The current finding extends previous literature in several ways. First, previous studies have already demonstrated that individuals with ADHD are uniquely characterized by inconsistent decision-making, as apparent in both choice and reaction time estimates [61, 62]. These studies further suggested that these behavioral inconsistencies in ADHD tend to wash out under conditions of immediate reward. For example, ADHD has been found to exhibit marked behavioral inconsistency in reaction times (i.e., increased RT variability) when performing sustained attention/executive function tasks (for review, see [59, 62, 63]). In a meta-analysis report, RT variability was further found to show the highest effect size for group differences. Thus, the mechanisms that contribute to this ADHD phenotype can be of much importance to how we understand ADHD. The sustained attention/executive functions tasks that reported increased RT variability in ADHD were mostly performed in the absence of any immediate reward or feedback. Remarkably, when immediate feedback or reward was delivered, ADHD demonstrated a significant improvement in RT variability estimates. Improved RT variability with the delivery of immediate reward was found in a variety of paradigms, including go/no-go [63], choice reaction time task [64, 65], simple reaction time [66], and time discrimination task [67, 68]. However, these studies are characterized almost exclusively by choice-feedback coupling, such that the outcome is given after a single choice and uses between-block manipulations (blocks with/without feedback). Here, we show both increased RT variability in ADHD compared to HC and the absence of group difference in RT variability estimates *within a single trial*. This is important since it refutes claims that a general motivation induced across a block is the sole reason for improved performance in ADHD under conditions of immediate reward/feedback. Instead, our findings point toward a more refined value-action mechanism that leads to increased inconsistency in ADHD.

Second, we find that only for first-stage choices ADHD show attenuated sensitivity to action values in terms of RT variability. Therefore, one explanation for both increased inconsistency in RTs in ADHD [59, 62, 63] and previous findings regarding the improvement in this estimate under immediate reward [63–66, 68] might be an attenuated ability to assign and follow internal action values. Specifically, when performing a task in the absence of immediate reward/feedback, the individual is required to hold in mind the overall value of task completion. This might lead to noisier action values in ADHD compared with HC, which in turn is reflected in noisier reaction times. Therefore, individuals need to hold an overall value of task completion to keep interest and guide the selection of accurate actions. Since individuals with ADHD have known deficits in reward neural processing and tend to discount future outcomes [68], they may encounter particular difficulty in assigning value to current actions based on a distant goal. Indeed, reinforcement learning studies have confirmed both in simulations and empirical studies that a reduced ability to assign value to actions leads to prolonged and more inconsistent RTs, a finding that we also replicated in the current study across groups. Therefore, we speculate that the process of internally assigning value to actions can be a major contributing mechanism to increased RT variability in ADHD.

Why do ADHD individuals exhibit noisier choices during first-stage choices? The current study cannot offer a clear explanation, yet we will discuss a few explanations. First, it might be that ADHD individuals are more explorative in their behavior [6, 25, 61, 69, 70]. Specifically, a few reinforcement learning studies suggested that less value-directed behavior in ADHD might be the result of the directed decision to explore rather than a reduced ability to follow action values. For example, Dubois et al. [69] examined explore/exploit decision-making among children in early and late adolescence. They found that participants with high scores in self-report symptoms of ADHD made more exploratory decisions. Next, Dubois & Hauser [71] further linked exploration and the impulsivity component within ADHD in an explore/exploit decision-making task. Addicott et al. [72] also examined explore/exploit decision-making using the 6-armed bandit task where, on each trial, six bandit options were depicted on a computer screen, and participants selected one and received a reward in various probabilities. They compared ADHD and HC groups of adults on explore/exploit decisions that were modeled using reinforcement learning algorithms. They found that ADHD participants made more exploratory decisions (i.e., chose options without the highest expected reward value) and earned fewer points than HC in all three study days. Frank et al. [25] examined go/no-go decision-making between ADHD and HC groups of young adults. They additionally used computational models in order to predict participants’ behavior. They found that ADHD participants were more likely to display inconsistent choices from trial to trial and that increased inconsistent behavior was correlated with increased variability in reaction times.

Another explanation of the reduced tendency to act according to value actions in first stage choice in ADHD might be the temporal distance from the outcome. That is, in the current task, we cannot rule out the fact that it is not the actual sequence or the fact that first-stage choices were initiatory actions in a sequence of three decisions. Rather, it might be that the temporal delay per se is the reason for the reduced ability to follow action values. We note that a previous study suggested that, counterintuitively, ADHD individuals improve their ability to learn from feedback when the action outcome epoch is prolonged [73]. However, dedicated studies should carefully disentangle temporal delay from task stages to account for this explanation.

Moreover, it is interesting to speculate the role of another cognitive process that was identified as associated with ADHD and the current finding. For example, the ability to assign and follow action values for initiatory actions might require executive functions such as working memory updating and sustained attention. Working memory plays a fundamental role in cognition, allowing one to hold information “in mind” and is considered a well-known attentional control system [40]. Since sequential learning consists of multiple stages that need to be learned, working memory capacity might be an important trait that affects this ability. Previous studies have highlighted individual differences in working memory capacity, demonstrating that lower capacity is associated with higher attentional deficits [36, 40, 74–78]. Furthermore, our findings may be explained by considering sustained attention, which pertains to an individual’s capacity to sustain focus on a task or stimulus over an extended period. Given the relatively prolonged duration of the multiple-stage task (approximately 50 min) and the demanding nature of the cognitive processes required to navigate its stages and outcomes, sustained attention emerges as a potentially influential factor affecting task performance. Difficulties in sustained attention are one of the core deficits in ADHD [79] and are reflected by increased omission errors [80, 81], lack of inhibitory control by an increased number of commission errors [80, 81], and unstable cognitive processing by increased reaction time variability [82] as shown using a continuous performance task (CPT). Hence, future studies can examine with a larger sample size the ability of working memory and/or sustain attention to moderate the group differences.

In terms of generalizing our results to the overall ADHD population, we note that our study was performed using young adults with/without ADHD. The sample was a clinical sample that included participants who sought treatment in the community, and as such, can be seen as part of the much-needed growing literature regarding ADHD in this age group [83]. However, it would be important to examine whether the observed attenuated sensitivity to initiatory action–value is also reflected in young children. Specifically, we speculate that since executive functions are only developing during early ages [84], and since these executive functions might take a role in action–value assignment, we should expect similar and even larger effects in children with ADHD compared to what was observed in young adults. Therefore, there is a need for further studies in different age groups that would also control for the moderation effect of different executive functions (i.e., working memory/response inhibition). Furthermore, our sample included only college students and, therefore, might show higher general cognitive abilities than the overall young adult population of the same age. Therefore, this might somewhat limit our ability to generalize our findings to the overall young adult population. However, IQ testing suggested that general cognitive abilities for both ADHD and HC groups are within the expected age norms and widely spread across the IQ range (see SI). Furthermore, we found no differences in IQ between the ADHD and HC groups. Therefore, we argue that despite being a college population, the current sample is not dramatically different from the overall young adult population.

Another point that should be considered in future studies is that deficits in value-based actions are not exclusive to ADHD and were also observed in certain impulsive disorders, including substance use disorders (SUD) and alcohol use disorders (AUD). Given the well-documented comorbidities between ADHD and SUD/AUD, perhaps attributable to their shared core symptoms of impulsivity [85, 86], we should consider the potential influence of these impulsive disorders on action–value processing effects [87, 88]. E.g., Sebold et al. [89] demonstrated that AUD is associated with alterations in value-based decision-making. Furthermore, in the context of SUD, Groman et al. [90] conducted a review and showed evidence suggesting that value updating following positive outcomes, but not negative outcomes, predicts escalation in SUD. However, given none of the participants were diagnosed with impulse disorders, and the self-report questionnaires assessing AUD and SUD yielded similar results between the groups, we can conclude that our results are not mainly driven by increased addiction/impulsive disorders in the experimental group.

Finally, a few limitations should be mentioned. First, a major limitation of this study pertains to the limited sample size. Specifically, the sample for this study comprised 54 participants, with 26 HC and 28 ADHD participants. Due to the possible inflated influence of small sample sizes on effect sizes that tend to characterize ADHD research [91], future studies with larger numbers of patients are required to confirm our results and conclusions. Notwithstanding, we employed Bayesian statistical methodologies in our analysis, a choice informed by the recognition that Bayesian approaches often exhibit reduced susceptibility to sample size constraints compared to classical frequentist statistical methods (For a review, see [92]) and allow to more delicately describe the uncertainty in the estimates. Second, our design does not allow us to differentiate whether ADHD individuals adopted different speed-accuracy response criteria in first-stage choices. In future work, integration of evidence accumulation model (e.g., DDM) parameters may also provide more clarity, as these models can provide estimates of choice sensitivity that are informed by both RT and accuracy [60, 93]. Third, our study cannot disentangle whether temporal distance from the outcome, unique properties of first-stage actions, or both are behind the effect, which should be examined using dedicated experimental manipulation in future research.

In conclusion, individuals with ADHD exhibit reduced choice accuracy and higher RT variability compared to HC individuals for first-stage, initiatory action but not for the third-stage, terminal action. Our computational modeling uncovered that individuals with and without ADHD assign and act upon internal action values in sequential decision-making tasks, and as shown here, there are differences between the groups in the ability to do so. This highlights the need to address and deeper understand the internal action values as an important mechanism in ADHD.

### Supplementary information

SI

## Data Availability

Data, regression analysis, and computational modeling analysis codes are publicly available at https://osf.io/ws47k/.
