# Supplementary material for: Exploring the steps of learning: computational modeling of initiatory-actions among individuals with attention-deficit/hyperactivity disorder
Source: Transl Psychiatry. 2024 Jan 8;14:10. doi: 10.1038/s41398-023-02717-7 (PMC10774270; doi:10.1038/s41398-023-02717-7)
Supplement: Supplementary file 1 — SI [file 41398_2023_2717_MOESM1_ESM.docx]

**Supporting information**

**Methods**

**Characteristics of ADHD and HC groups.** Overall, as can be seen in Figure S1, we did not find any substantial differences in estimates of depression (BDI), OCD (OCIR), anxiety (STAI), autism (AQ), IQ (Raven’s), alcohol abuse (AUDIT), or cannabis abuse (CUDIT) between HC and ADHD groups.


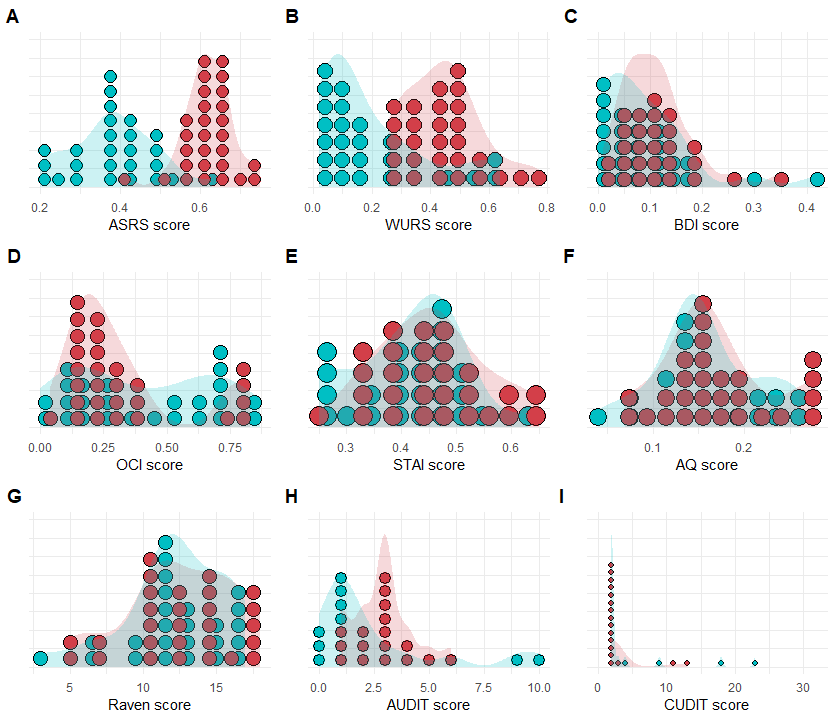


**Figure S1.** **Characteristics of ADHD and HC groups.** Scattered plots for each self-report questionnaire by group. (**A**) ASRS - Adult ADHD Self Report Scale, (**B**) WURS - Wender Utah Rating Scale for the Attention Deficit Hyperactivity, (**C**) BDI - Beck Depression Inventory, (**D**) OCI - Obsessive-Compulsive Inventory, (**E**) STAI - State Trait Anxiety Inventory, (**F**) AQ - Autism Spectrum Quotient, (**G**) Raven - Raven Progressive Matrices (**H**) AUDIT - Alcohol Use Disorders Identification Test, (**I**) CUDIT - Cannabis Use Disorders Identification Test. Overall, we found significant difference only in ADHD symptoms.

**IQ differences between groups.** We wanted to examine whether there is a difference in general cognitive abilities between ADHD and HC groups (see Figure S2). For that aim we converted the Raven scores to standardized IQ scores according to the Raven’s norms^1^. We performed an independent t-test between the IQ scores of HC (M = 105.00, SD =14.24) and ADHD (M = 107.75, SD = 16.12) groups, and found no significant difference [t(52) = -0.66, CI_95%_ between -11.12 to 5.62, *p* = .51].


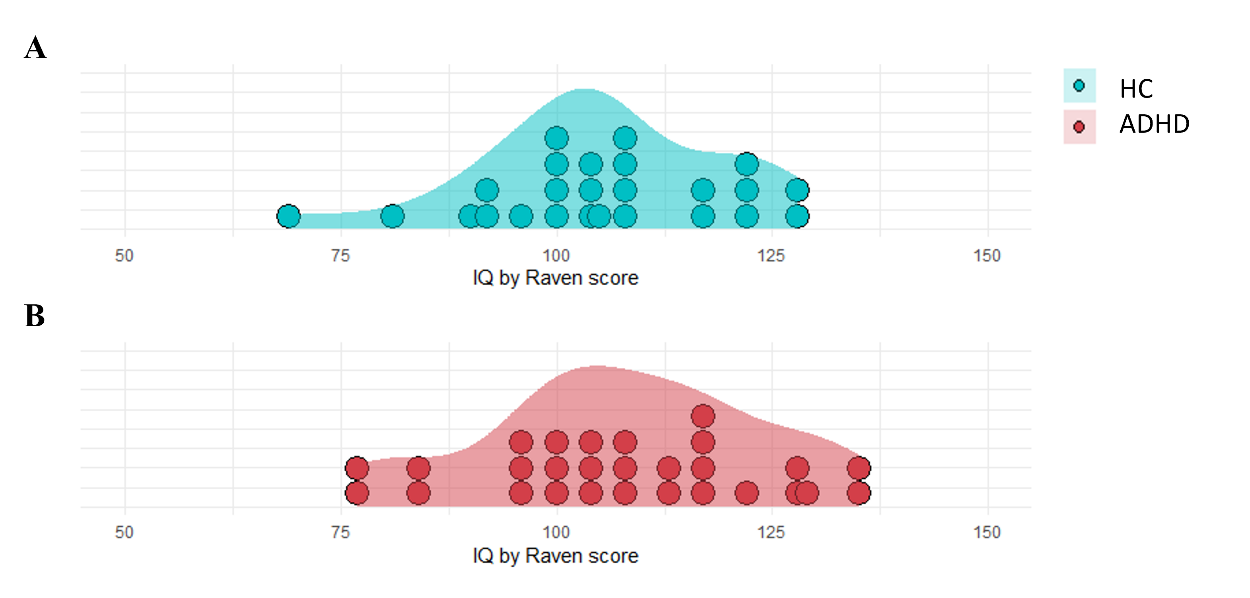


**Figure S2.** **Intelligence Quotient for the ADHD and HC groups**. We estimated IQ using the Raven advance matrix test (normalized using Raven’s age norms). IQ scores were found to be widely spread across the IQ range for both HC (**A**) and ADHD (**B**) groups with no significant differences (independent t-test p=.51).

**Reinforcement learning task.** At the start of the task, participants were presented with the task instructions including the stimuli structure, completed a short practice, and completed a multichoice quiz which they had to complete with 100% accuracy. Participants were told that they need to do their best to find the puppy as many times as possible. Participants completed two blocks, with 100 trials each and were allowed a self-paced rest every 50 trials. Participants received a new set of images (i.e., houses, doors, chests) in each block that were counterbalanced between subjects. Each trial starts with a fixation (1000ms), followed by the presentation of two houses. Participants then choose a house freely using a right/ left corresponding response-key press (‘s’ or ‘k’ keys in a QWERTY keyboard; until response with 6sec deadline). After making a choice, a squared placeholder located around the chosen stimuli (house, door, or chest) and the unchosen stimulus disappeared to allow choice feedback (500ms). At the end of the third stage the outcome displays (1 or 0, finding hidden puppy or not;1sec).

**Choice accuracy and difficulty.** Choice accuracy was defined differently for each stage. Specifically, for choices in first and second stages, this means that an accurate choice should be defined as a choice in the house/door that led to the chest with the highest true expected value (i.e., best sequence). For the third stage, an accurate choice was simply defined as a choice in the chest with the highest expected value between the offered pair. Similarly, choice difficulty was also defined differently for each stage. Specifically, for first and second stages ‘choice difficulty’ was defined as the absolute difference in the maximum expected value that can be obtained following a choice in each of two offered actions. For third stage actions, choice difficulty was the absolute difference in the chests’ true expected values.

**Bayesian parameters estimation.** Bayesian logistic regression and reinforcement learning computational modeling analyses were performed using 'brms', ‘rstan’, and ‘loo’ packages in R ^2–4^. Reinforcement learning modeling was performed using the ‘cmdstanr’. All models included population level (fixed effects) and participants level (random effects) parameters for all estimated models and sampled with weakly informative priors (We further tested each regression model with narrow and wider priors to make sure the conclusion has not changed). For contrasts estimation we used ‘emmeans’ package in R ^5^. For exGaussian analysis we used the ‘brms’ and ‘stan’ exGaussian likelihood estimation ^2,6,7^. All chains were visually examined using trace plots, pairs plots, and R-hat estimates and found to show good chain mixing. We report the median, 89% and 95% equal-tailed density interval (HDI) ^8^, and probability of direction for parameters’ posterior distributions (for logistic regression estimates are on the log-odds scale ^9^). We further referenced in each result a figure panel in the SI that depicts the full posterior (see Figure S5-8). Note that 89% HDI were chosen along with the recommendation made by McElreath 2020 ^8^. This refers to the fact that there is nothing special in the 89% CI value (or any other). 89% is convenient since it is a prime number that is easy to remember and compute. Therefore, in this work we make an effort to avoid hypothesis testing and instead describe the posterior distributions to a full extent where possible.

**Using ex-Gaussian distribution to estimate RT variability.** The ex-Gaussian distribution has three parameters: μ represents the mean of the normal component, σ represents the standard deviation of the normal component, and τ is the parameter that describes the tail of the distribution. The ex-Gaussian distribution is a useful statistical model for analyzing RT data in cognitive science and specifically in ADHD research, due to its ability to describe the tail of the distribution with a single parameter (τ). We made use of the ‘brms’ ^2^ ex-Gaussian likelihood function and estimated hierarchical Bayesian regression where the effect of reported predictors was assessed on both μ and τ, including an intercept parameter for sigma. For each reported regression using ex-Gaussain distribution we fitted the regression to both μ and τ estimates. Given our interest in RT variability, we reported only the findings regarding the τ estimates (see SI for complete analysis with all exGaussian parameters). Note that in our design, exGaussian analysis was performed independently of the reinforcement learning modeling that allowed us to estimate trial-by-trial action values according to participants choices and rewards history. Therefore, any associations between value estimation and exGaussian RT analysis is strictly due to the nature of the observations and cannot be a confound of the modeling approach. Coefficients for tau are reported on the log-scale.

**Priors.** Bayesian regression analysis was performed using appropriate weakly informative priors. First, Bayesian logistic regression analysis, that was performed in order to estimate accuracy rates, we used a student t distribution with 6 degrees of freedom, mean of 0 and standard deviation of 2.5. This prior was examined and selected due to several literature recommendations ^10,11^. Second, for the ex-gaussian regression analysis that was performed in order to estimate reaction time variability, we set three different priors for the mean (normal distribution with mean of 0.65 and standard deviation of 0.1), sigma (normal distribution with mean of -3 and standard deviation of 0.5) and τ (normal distribution with mean of -1.20 and standard deviation of 0.5).

**Parameter recovery analysis.** To ensure that the model can be used adequately to estimate latent parameters, we performed a parameter recovery analysis. Specifically, we simulated 100 agents performing the same task, with the same number of trials as the empirical task. Agents’ parameters were simulated hierarchically from a gaussian distribution [𝛼_1_, 𝛼_2_, 𝛼_3_, mean=0.3, sd=1; $\beta$_1_,$\beta$_2,_$\beta$_3,_ mean=4, sd=1.5; 𝜆, mean=0.5, sd=1]. We then sampled and updated the prior distribution of each parameter based on the artificial data. Examining both population (i.e., fixed effects) and individual (i.e., random effects) posterior distributions indicated excellent parameter recovery with no indication of bias (See Figure S3).

**
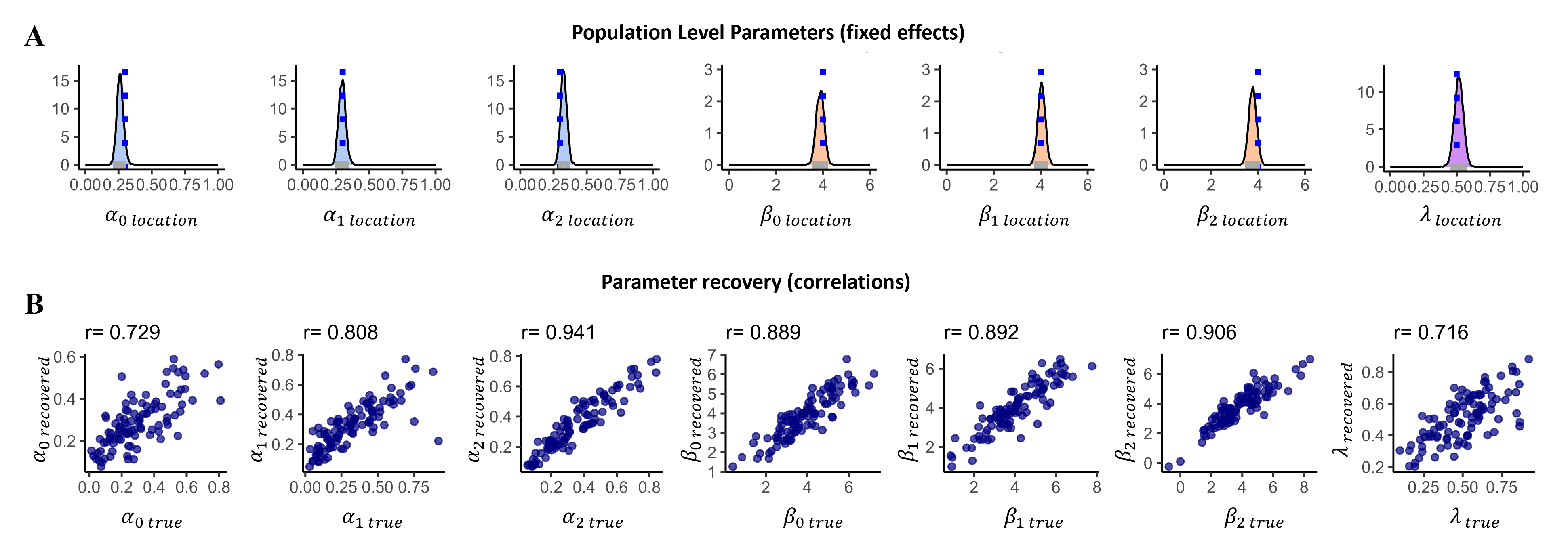
**

**Figure S3.** **Parameter recovery for the reinforcement learning model used to estimate value-based learning.** Specifically, 100 agents were simulated playing the task with varying parameters generated using hierarchical sampling. (**A**) Population level parameters (fixed effects) with the true parameter in dashed blue line and the recovered posterior. (**B**) Individual level parameters plotted against the mean posterior parameter distribution of each individual. Overall, these results show excellent parameter recovery for the model.

**Model posterior sampling.** Overall, the model included seven free population level effects, along with their individual-level random effects. We sampled the posterior of each parameter hierarchically (fixed and individual-level random effects), and separately for each group using ‘stan’ ^6,7^. For all parameters we used a weakly informative prior and all parameters were sampled using a non-centered parameterization, with a logit link for learning-rates and lambda parameters. To sample from the posterior we used 4 mcmc chains over 3000 iterations from which 2000 were used as warmups. We examined chain convergence, rhat, and effective sample size to ensure that the sampling converged to the parameters posteriors ^7,8^.

**Results**

**Accuracy rates across groups analysis.** First, we sought to show that participants have learned to make choices according to the latent true expected values of the options in the task. We defined an accurate choice, as a choice in the action that had the higher true expected value (for first and second stage choices, we used the highest expected value that could be reached, see Methods for details). We then perform a hierarchical logistic Bayesian regression analysis to predict response accuracy (0 vs. 1) using an intercept only model. We found evidence for above-chance accuracy rates, suggesting overall participants were able to learn to hidden latent values of each action [54% CI_89%_ between .53 to .57; Intercept median = 0.18 CI_89%_ between 0.12 to 0.25, pd ~ 100%]. We then extended this analysis and calculated a ’choice difficulty‘ variable quantifying the absolute difference between the expected values of the two offered actions (see Methods). We examined the association between choice difficulty and accuracy rates, expecting to see an increase in accuracy rates when the difference between the expected values was higher. We repeated the hierarchical Bayesian regression predicting choice accuracy using both an intercept and choice difficulty as predictor and found a substantial increase in response accuracy when choice difficulty was easier [slope posterior median = 0.88 CI_89%_ between 0.40 to 1.37, probability of direction (pd) = 99.6%], showing that a change of 0.5 in the difference between the true expected value of the two offered actions was associated with an increase of 10.8% in accuracy rates. This analysis suggests that participants were able to make accurate choices according to the true expected values defined by the experimenter. Furthermore, the fact that accuracy rates changed as a function of the difference between the true expected values of the two offered options gives strong support to the conclusion that overall, participants were able to perform the task well.

**Accuracy rates and IQ – group differences at the first stage.** To examine whether IQ influences on the difference in performance between ADHD and HC participants on accuracy rates at the first stage we conducted a Bayesian regression analysis where we predicted choice accuracy (0/1 for choosing the offer with the lower/higher true expected value, respectively), using choice difficulty on the first stage (absolute difference between the true expected values of the two offers), centered IQ score and group (HC vs. ADHD). When examining the interaction between group and choice difficulty on the first stage, we found that the effect of choice difficulty on accuracy rates was smaller for ADHD compared to HC [posterior median of the groupXchoice-diffculty on first stage effect = -1.70 CI_89%_ between -3.13 to -0.16, pd = 96.50%]. However, when examining the centered IQ score effects (IQ score effect, IQ score x choice-difficulty interaction, IQ score x group interaction, IQ score x choice difficulty x group interaction) we found no significant influence on the accuracy rates (See Figure S4 and Table S1 for coefficients table).


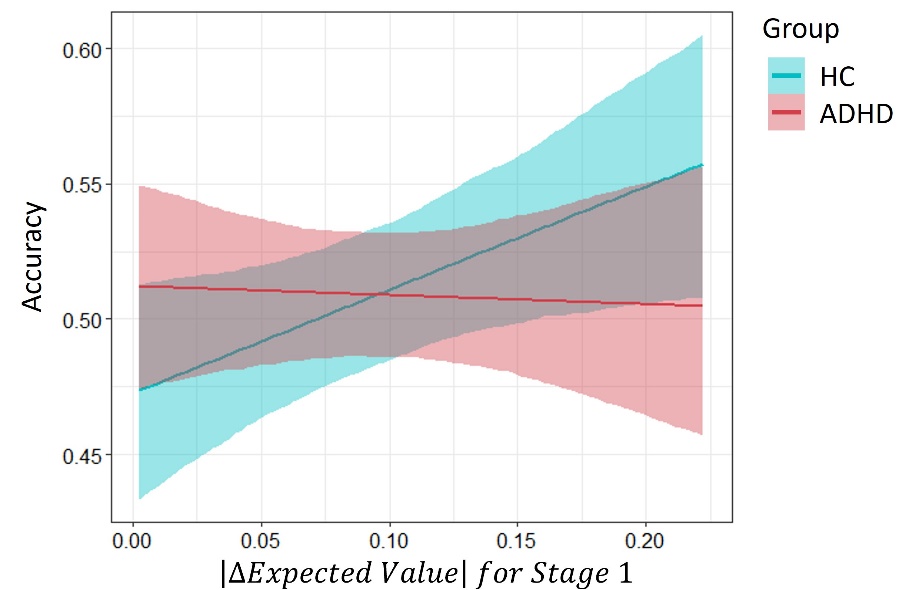


**Figure S4**. Choice accuracy on the first stage on the first stage with group (HC and ADHD), centered IQ score and the difference in the choices true expected values as predictors. ADHD individuals showed an attenuated sensitivity to choice difficulty changes, importantly centered IQ score did not affect this effect.

| *Table S1.* Choice accuracy on the First Stage coefficients for a model estimating choice accuracy on the first stage with Group, centered IQ score and the difference in the choices true expected values as predictors. | | | |
| --- | --- | --- | --- |
| Parameter | Median | CI_89%_ | pd |
| Intercept | -0.11 | [-0.24, 0.02] | 91.80% |
| Difference in the choices true expected values for Stage 1 | 1.53 | [0.45, 2.59] | 98.45% |
| Centered IQ score effect | -0.007 | [-0.66, 0.66] | 50.85% |
| ADHD Group effect | 0.16 | [-0.02, 0.33] | 92.00% |
| Difference in the choices true expected values X centered IQ score | -0.93 | [-5.25, 2.70] | 64.80% |
| Difference in the choices true expected values X ADHD Group for Stage 1 | -1.70 | [-3.13, -0.16] | 96.50% |
| Centered IQ score X ADHD Group for Stage 1 | -0.56 | [-1.62, 0.50] | 79.75% |
| Difference in the choices true expected values X centered IQ score X ADHD Group | 0.86 | [-7.31, 8.77] | 57.00% |
| *Note. The Group variable was treated as factors, where the variable 'ADHD Group' refers to the ADHD group that was coded as 1 (and HC group was coded as 0). CI – credible interval, PD - probability of direction.* | | | |

**Accuracy rates - group difference analysis.**

**
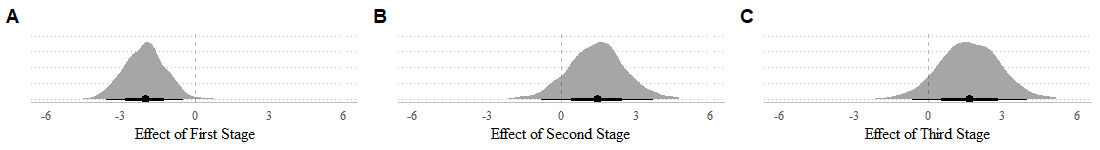
**

**Figure S5**. Posterior density for the coefficients of the choice accuracy with choice difficulty, stage and group as predicators. (**A**) Overall, we found group difference on the First stage but not on the (**B**) Second and (**C**) Third stages.

**Reaction-time variability across groups analysis**. RT variability was previously found to be associated with the value difference between actions ^12,13^. We first sought to replicate previous reports and demonstrate across groups and stages that RT variability is larger when it was easier for participants to make value-based choices. We therefore examined the association between choice difficulty (absolute difference between the true expected values of the two offers, see Methods) and RT variability (indexed using the well-established τ parameter in an ex-Gaussian distribution, see Methods). We performed a hierarchical Bayesian linear regression predicting the τ parameter with choice difficulty. As expected, we found a negative association so that a change of 0.5 in the difference between the expected value of the two offered choices, resulted in a decrease of 89ms in the slow tail of the RT distribution [posterior median for the slope effect on τ parameter = -0.53 CI_89%_ between -0.67 to -0.38, pd ~ 100%].

**Reaction-time variability - group difference analysis.**


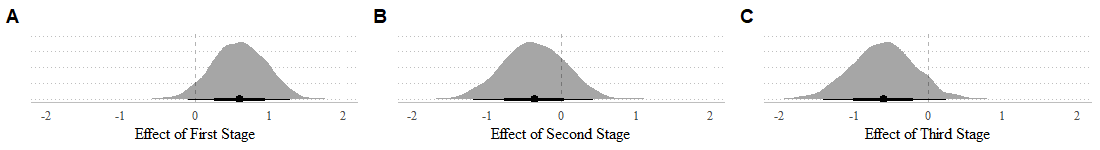


**Figure S6**. Posterior density for the coefficients of the RT variability with choice difficulty, stage and group as predicators. (**A**) Overall, that the effect of choice difficulty on RT variability was smaller for HC compared to ADHD in First stage choices. (**B**) However, for the Second and (**C**) Third stage this effect reversed with a slightly larger effect of choice difficulty on RT variability estimates for ADHD compared to HC.

| *Table S2.* Reaction-time variability coefficients for a model estimating reaction-time using an ex-Gaussian distribution likelihood with Group, Stage and the difference in the choices true expected values as predictors. | | | | |
| --- | --- | --- | --- | --- |
| Parameter | Median | CI_89%_ | CI_95%_ | pd |
| **Mu parameter coefficients:** |  |  |  |  |
| Intercept (The effect for Stage 1) | 0.400 | [ 0.38, 0.43] | [0.37, 0.44] | 100% |
| Stage 1 to 2 | 0.002 | [-0.02, 0.02] | [-0.02, 0.03] | 57.10% |
| Stage 2 to 3 | -0.007 | [-0.03, 0.01] | [-0.03, 0.02] | 69.95% |
| Difference in the choices true expected values for Stage 1 | 0.060 | [ 0.00, 0.12] | [-0.01, 0.14] | 94.30% |
| ADHD Group effect for Stage 1 | -0.020 | [-0.06, 0.01] | [-0.07, 0.02] | 83.20% |
| Stage 1 to 2 X Difference in the choices true expected values | -0.070 | [-0.14, 0.00] | [-0.15, 0.01] | 95.70% |
| Stage 2 to 3 X Difference in the choices true expected values | -0.030 | [-0.10, 0.04] | [-0.11, 0.05] | 73.25% |
| Stage 1 to 2 X ADHD Group | -0.030 | [-0.05, 0.00] | [-0.06, 0.01] | 92.15% |
| Stage 2 to 3 XADHD Group | -0.004 | [-0.03, 0.02] | [-0.04, 0.03] | 60.35% |
| Difference in the choices true expected values X ADHD Group for Stage 1 | -0.020 | [-0.11, 0.07] | [-0.13, 0.08] | 63.80% |
| Stage 1 to 2 X Difference in the choices true expected values X ADHD Group | 0.070 | [-0.02, 0.17] | [-0.05, 0.19] | 88.65% |
| Stage 2 to 3 X Difference in the choices true expected values X ADHD Group | 0.020 | [-0.08, 0.12] | [-0.11, 0.14] | 60.60% |
| **Sigma parameter coefficients (log-scale):** Intercept | -2.790 | [-2.82, -2.76] | [-2.28, -2.76] | 100% |
| **Tau parameter coefficients (log-scale):** Intercept (The effect for Stage 1) | -1.150 | [-1.28, -1.01] | [-1.32, -0.97] | 100% |
| Stage 1 to 2 | 0.150 | [ 0.02, 0.26] | [0.00, 0.29] | 97.15% |
| Stage 2 to 3 | 0.160 | [ 0.05, 0.27] | [0.03, 0.29] | 99.10% |
| Difference in the choices true expected values for Stage 1 | 0.040 | [-0.37, 0.46] | [-0.46, 0.54] | 55.35% |
| ADHD Group effect for Stage 1 | 0.150 | [-0.04, 0.35] | [-0.09, 0.39] | 90.80% |
| Stage 1 to 2 X Difference in the choices true expected values | -0.940 | [-1.44, -0.46] | [-1.53, -0.36] | 99.90% |
| Stage 2 to 3 X Difference in the choices true expected values | -0.320 | [-0.80, 0.18] | [-0.90, 0.29] | 85.25% |
| Stage 1 to 2 X ADHD Group | 0.030 | [-0.14, 0.18] | [-0.18, 0.22] | 59.25% |
| Stage 2 to 3 X ADHD Group | -0.110 | [-0.26, 0.03] | [-0.30, 0.07] | 87.90% |
| Difference in the choices true expected values X ADHD Group for Stage 1 | 0.600 | [ 0.03, 1.16] | [-0.08, 1.28] | 95.25% |
| Stage 1 to 2 X Difference in the choices true expected values X ADHD Group | -0.370 | [-1.05, 0.29] | [-1.19, 0.43] | 80.00% |
| Stage 2 to 3 X Difference in the choices true expected values X ADHD Group | -0.600 | [-1.28, 0.05] | [-1.41, 0.24] | 92.35% |
| *Note. The parameters were taken from ex-Gaussian distribution, Stage and Group were treated as factors, where the variable 'ADHD Group' refers to the ADHD group that was coded as 1 (and HC group was coded as 0). As for the Stage factor - Stage 1 was coded as 0, the additional given by Stage 2 described as Stage 1 to 2 and the additional given by Stage 3 described as Stage 2 to 3. CI – credible interval, PD - probability of direction.* | | | | |

**Computational reinforcement learning modeling.** The model allowed us to gain and estimate for the internal values for each action given the best estimated individual parameters, and the choice-outcome history of each individual ^14–18^. We report our modeling approach extensively in the Methods section. However, to gain some intuition of the modeling approach, one can describe these latent values (also sometimes referred as Q-values in reinforcement learning) as a personalized summary statistics of the rewards that followed a certain choice. This summary statistics is ‘personalized’ in the sense that it takes into account the specific outcome history that preceded a certain trial, take into account a discounting over trial (outcomes of distant trials influence less, accounted by an alpha parameter), discounting over stages (outcome affect first stages less compared with third, accounted by a lambda parameter), and overall noise (tendency to prefer the higher value option, accounted by a beta parameter). After obtaining these internal value estimates, we repeated the same regression analyses that we performed before, examining the association of internal action values on choice accuracy and RT variability in each group.

**Model fitting.** We fitted the model to each group separately using hierarchical Bayesian parameter estimation. We further performed a nested model comparison and found that a saturated seven parameter model had the best fit to the empirical data (For details see *nested model comparisons* in SI). When comparing the population level (fixed effects) parameters of the two groups, we found substantial group differences only in the tendency to follow latent action values in first stage choices ($\beta$ parameter; see Table S3). This corresponds well with our previous analysis regarding accuracy rates and suggests that overall ADHD were less consistent in acting according to outcome history when making first stage choice. However, this was not observed in second and third stage choices.

| *Table S3.* Posterior parameter distribution across groups for the computational model reinforcement learning parameters | | | | | | |  |
| --- | --- | --- | --- | --- | --- | --- | --- |
|  | **Healthy control** | | **ADHD** | | **Differences** | | |
|  | Mean | CI_89%_ | Mean | CI_89%_ | Mean | CI_89%_ | pd |
| 𝛼_1_ | 0.66 | [0.39, 0.97] | 0.85 | [0.72, 1.00] | 0.19 | [-0.14, 0.55] | .81 |
| 𝛼_2_ | 0.39 | [0.14, 0.63] | 0.26 | [0.03, 0.51] | -0.13 | [-0.51, 0.26] | .72 |
| 𝛼_3_ | 0.10 | [0.02, 0.18] | 0.11 | [0.03, 0.19] | 0.01 | [-0.12, 0.14] | .58 |
| $\beta$**_1_** | **4.40** | **[3.07, 5.76]** | **2.20** | **[1.07, 3.39]** | **-2.20** | **[-3.84, -0.31]** | **.98** |
| $\beta$_2_ | 4.72 | [3.07, 6.33] | 5.30 | [3.66, 7.12] | 0.58 | [-1.79, 2.97] | .66 |
| $\beta$_3_ | 4.12 | [3.16, 5.05] | 4.08 | [2.88, 5.32] | -0.03 | [-1.58, 1.43] | .51 |
| 𝜆 | 0.51 | [0.18, 0.86] | 0.65 | [0.32, 1.00] | 0.14 | [-0.35, 0.67] | .68 |
|  |  |  |  |  |  |  |  |

**Nested model comparisons.** It is possible that participants might have been using a simpler model then the one we specified (for example, one learning rate / temperature parameter for all stages). We therefore fitted two reinforcement learning models that differentiated in the number of parameters: a baseline model with three parameters (𝛼, $\beta$ and 𝜆) and a comparison model with seven parameters (one 𝛼 for each stage, one $\beta$ for each stage, and 𝜆). We performed model comparison using widely applicable information criteria (WAIC) to assess the relative fit of two competing models to the data of each group (HC, ADHD). The models were fitted using the ‘cmdstanr‘ software package (version 0.5.3) in R (version 4.2.3), and the WAIC values were calculated using the loo package (version 2.6.0). First, the WAIC values of the HC group were calculated. The baseline model and the seven-parameters model WAIC values of the HC group were 16849.4 (SE = 100.8) and 16633.5 (SE = 102.2), respectively. The difference in WAIC values between the two models was 107.9 (SE = 18.6), and the weights given to every model (baseline model: 0.154, seven-parameters model: 0.846) indicating that the seven-parameters model had a better fit to the data than the baseline model even after penalizing for the additional number of parameters. Next, for the ADHD group, the WAIC values for the baseline model and the seven-parameters model were 17969.1 (SE = 103.0) and 17652.2 (SE = 106.1), respectively. The difference in WAIC values between the two models was 158.4 (SE = 21.6), and the weights given to every model (baseline model: 0.147, seven-parameters model: 0.853) indicating that the seven-parameters model had a better fit to the data than the baseline model for the ADHD group as well. Overall, the results of the WAIC model comparison indicate that the seven-parameters model had a better fit to the data than the baseline model, for both HC and ADHD groups, and was therefore preferred.

**Accuracy rates estimated using internal action values.**

**
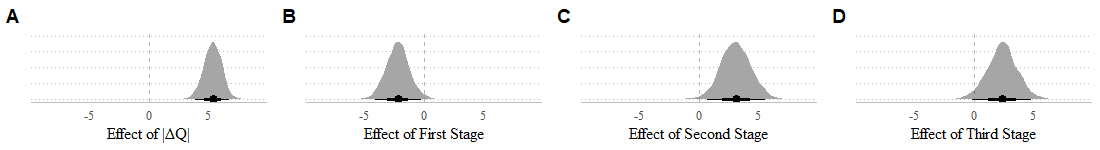
**

**Figure S7.** Posterior density for the coefficients of the choice accuracy with absolute difference in internal value, stage and group as predicators. (**A**) We found effect for absolute difference in internal value, such that higher Q-value differences were coupled with higher choice accuracy across stages and groups. (**B**) A substantial group x |ΔQ| interaction for the First stage was found such that ADHD exhibited lower sensitivity to Q-values difference in terms of choice accuracy compared with HC. (**C**) A tendency toward a group x |ΔQ| interaction in the positive direction for the Second and (**D**) Third stages. Overall, these findings resonated with the finding that ADHD had lower decision noise parameter at Stage 1 compared to their peers.

**Reaction-time variability as a function of internal action values.**


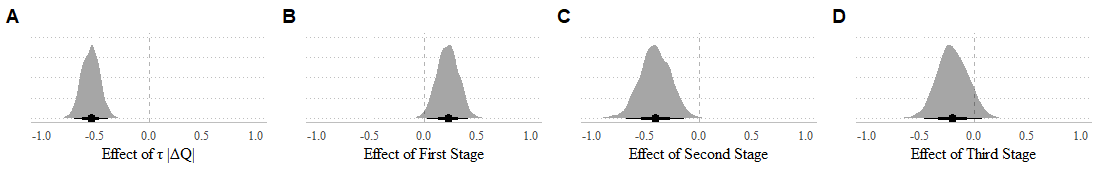


**Figure S8**. Posterior density for the coefficients of the choice accuracy with absolute difference in internal value, stage and group as predicators. (**A**) We found effect for absolute difference in internal value, such that higher Q-value differences were coupled with lower τ values across stages and groups. (**B**) A substantial group x |ΔQ| interaction for the First stage was found such that ADHD exhibited sensitivity to Q-values difference in terms of 𝜏 estimates compared with HC. (**C**) The effect reversed on the Second stage with a significant effect of Q-value difference on RT variability estimates for ADHD compared to HC. (**D**) A slightly smaller effect of Q-value difference on RT variability estimates for ADHD compared to HC.

| *Table S4.* Reaction-time variability coefficients for a model estimating reaction-time using an ex-Gaussian distribution likelihood with Group, Stage and the difference in internal values (\|ΔQ\|) as predictors. | | | | |
| --- | --- | --- | --- | --- |
| Parameter | Median | CI_89%_ | CI_95%_ | pd |
| **Mu parameter coefficients:** |  |  |  |  |
| Intercept (The effect for Stage 1) | 0.400 | [ 0.38, 0.42] | [0.37, 0.43] | 100% |
| Stage 1 to 2 | -0.001 | [-0.02, 0.02] | [-0.02, 0.02] | 52.20% |
| Stage 2 to 3 | -0.009 | [-0.03, 0.01] | [-0.03, 0.01] | 77.80% |
| Difference in the internal values for Stage 1 | 0.030 | [ 0.01, 0.05] | [0.01, 0.05] | 99.70% |
| ADHD Group for Stage 1 | -0.030 | [-0.06, 0.01] | [-0.06, 0.01] | 89.85% |
| Stage 1 to 2 X Difference in the internal values | -0.020 | [-0.05, 0.00] | [-0.05, 0.0] | 95.05% |
| Stage 2 to 3 X Difference in the internal values | 0.010 | [-0.01, 0.04] | [-0.01, 0.04] | 81.95% |
| Stage 1 to 2 X ADHD Group | -0.010 | [-0.03, 0.01] | [-0.04, 0.02] | 74.05% |
| Stage 2 to 3 X ADHD Group | 0.001 | [-0.02, 0.03] | [-0.03, 0.03] | 53.00% |
| Difference in the internal values X ADHD Group for Stage 1 | 0.009 | [-0.01, 0.03] | [-0.02, 0.04] | 73.00% |
| Stage 1 to 2 X Difference in the internal values X ADHD Group | 0.010 | [-0.02, 0.04] | [-0.03, 0.05] | 73.65% |
| Stage 2 to 3 X Difference in the internal values X ADHD Group | -0.020 | [-0.05, 0.01] | [-0.06, 0.02] | 86.75% |
| **Sigma parameter coefficients (log-scale):** |  |  |  |  |
| Intercept | -2.790 | [-2.82, -2.77] | [-2.82, -2.76] | 100% |
| **Tau parameter coefficients (log-scale):** |  |  |  |  |
| Intercept (The effect for Stage 1) | -1.040 | [-1.17, -0.91] | [-1.20, -0.88] | 100% |
| Stage 1 to 2 | -0.080 | [-0.16, 0.01] | [-0.19, 0.03] | 91.90% |
| stage 2 to 3 | 0.160 | [ 0.05, 0.26] | [0.03, 0.29] | 99.10% |
| Difference in the internal values for Stage 1 | -0.540 | [-0.67, -0.41] | [-0.70, -0.38] | 100% |
| ADHD Group for Stage 1 | 0.200 | [ 0.01, 0.37] | [-0.03, 0.41] | 95.30% |
| Stage 1 to 2 X Difference in the internal values | -0.110 | [-0.28, 0.06] | [-0.33, 0.11] | 85.15% |
| Stage 2 to 3 X Difference in the internal values | -0.250 | [-0.43, -0.09] | [-0.47, -0.04] | 99.30% |
| Stage 1 to 2 X ADHD Group | 0.060 | [-0.06, 0.18] | [-0.09, 0.21] | 80.00% |
| Stage 2 to 3 X ADHD Group | -0.140 | [-0.30, 0.01] | [-0.33, 0.04] | 93.85% |
| Difference in the internal values X ADHD Group for Stage 1 | 0.230 | [ 0.07, 0.38] | [0.03, 0.41] | 99.05% |
| Stage 1 to 2 X Difference in the internal values X ADHD Group | -0.410 | [-0.63, -0.18] | [-0.68, -0.14] | 99.90% |
| Stage 2 to 3 X Difference in the internal values X ADHD Group | -0.200 | [-0.42, 0.03] | [-0.46, 0.08] | 91.20% |
| *Note. The parameters were taken from ex-Gaussian distribution, Stage and Group were treated as factors, where the variable 'ADHD Group' refers to the ADHD group that was coded as 1 (and HC group was coded as 0). As for the Stage factor - Stage 1 was coded as 0, the additional given by Stage 2 described as Stage 1 to 2 and the additional given by Stage 3 described as Stage 2 to 3. CI – credible interval, PD - probability of direction.* | | | | |

**SI References**

1 Raven J, Raven JC, Court JH. *Manual for Raven’s progressive matrices and vocabulary scales*. Pearson: San Antonio, TX, 1998.

2 Bürkner P-C. Advanced Bayesian Multilevel Modeling with the R Package brms. *R J* 2018; **10**: 395–411.

3 Carpenter B, Gelman A, Hoffman MD, Lee D, Goodrich B, Betancourt M *et al.* Stan: A Probabilistic Programming Language. *J Stat Softw* 2017; **76**: 1–32.

4 Vehtari A, Gelman A, Gabry J. Practical Bayesian model evaluation using leave-one-out cross-validation and WAIC. *Stat Comput* 2017; **27**: 1413–1432.

5 Lenth RV, Bolker B, Buerkner P, Giné-Vázquez I, Herve M, Jung M *et al.* emmeans: Estimated Marginal Means, aka Least-Squares Means. 2023.https://cran.r-project.org/web/packages/emmeans/index.html (accessed 1 Jun2023).

6 Stan Development Team. RStan: the R interface to Stan. R package. 2020.http://mc-stan.org/.

7 Stan Development Team. Stan Modeling Language Users Guide and Reference Manual. 2022.https://mc-stan.org.

8 McElreath R. *Statistical rethinking : a Bayesian course with examples in R and Stan.* Second edition. Boca Raton : CRC Press, 2020.

9 Makowski D, Ben-Shachar MS, Chen SHA, Lüdecke D. Indices of Effect Existence and Significance in the Bayesian Framework. *Front Psychol* 2019; **10**.https://www.frontiersin.org/articles/10.3389/fpsyg.2019.02767 (accessed 7 Oct2022).

10 Gelman A, Jakulin A, Pittau MG, Su Y-S. A weakly informative default prior distribution for logistic and other regression models. *Ann Appl Stat* 2008; **2**. doi:10.1214/08-AOAS191.

11 Ghosh J, Li Y, Mitra R. On the Use of Cauchy Prior Distributions for Bayesian Logistic Regression. 2015. doi:10.48550/ARXIV.1507.07170.

12 Fontanesi L, Gluth S, Spektor MS, Rieskamp J. A reinforcement learning diffusion decision model for value-based decisions. *Psychon Bull Rev* 2019; **26**: 1099–1121.

13 Shahar N, Hauser TU, Moutoussis M, Moran R, Keramati M, NSPN consortium *et al.* Improving the reliability of model-based decision-making estimates in the two-stage decision task with reaction-times and drift-diffusion modeling. *PLOS Comput Biol* 2019; **15**: e1006803.

14 Botvinick MM. Hierarchical reinforcement learning and decision making. *Curr Opin Neurobiol* 2012; **22**: 956–962.

15 Dezfouli A, Lingawi NW, Balleine BW. Habits as action sequences: hierarchical action control and changes in outcome value. *Philos Trans R Soc B Biol Sci* 2014; **369**: 20130482.

16 Dezfouli A, Balleine BW. Actions, action sequences and habits: evidence that goal-directed and habitual action control are hierarchically organized. *PLoS Comput Biol* 2013; **9**: e1003364.

17 Dolan RJ, Dayan P. Goals and Habits in the Brain. *Neuron* 2013; **80**: 312–325.

18 Sutton RS, Barto A. *Reinforcement learning: an introduction*. Second edition. The MIT Press: Cambridge, MA London, 2018.
